# Chemical array system, a platform to identify novel hepatitis B virus entry inhibitors targeting sodium taurocholate cotransporting polypeptide

**DOI:** 10.1038/s41598-018-20987-w

**Published:** 2018-02-09

**Authors:** Manabu Kaneko, Yushi Futamura, Senko Tsukuda, Yasumitsu Kondoh, Tomomi Sekine, Hiroyuki Hirano, Kento Fukano, Hirofumi Ohashi, Wakana Saso, Ryo Morishita, Satoko Matsunaga, Fumihiro Kawai, Akihide Ryo, Sam-Yong Park, Ryosuke Suzuki, Hideki Aizaki, Naoko Ohtani, Camille Sureau, Takaji Wakita, Hiroyuki Osada, Koichi Watashi

**Affiliations:** 1 Department of Virology II, National Institute of Infectious Diseases, Tokyo, 162-8640 Japan; 2 Department of Applied Biological Sciences, Tokyo University of Science, Noda, 278-8510 Japan; 3 Chemical Biology Research Group, RIKEN Center for Sustainable Resource Science (CSRS), Wako, 351-0198 Japan; 4 Micro-signaling Regulation Technology Unit, RIKEN Center for Life Science Technologies (CLST), Wako, 351-0198 Japan; 5 Bio-Active Compounds Discovery Research Unit, RIKEN CSRS, Wako, 351-0198 Japan; 6 Chemical Resource Development Research Unit, RIKEN CSRS, Wako, 351-0198 Japan; 7 Department of Analytical Biochemistry, Meiji Pharmaceutical University, Kiyose, 204-8588 Japan; 8 The Institute of Medical Science, The University of Tokyo, Tokyo, 108-8639 Japan; 9 CellFree Sciences Co., Ltd, Matsuyama, 790-8577 Japan; 10 Department of Microbiology, Yokohama City University Graduate School of Medicine, Yokohama, 236-0027 Japan; 11 Drug Design Laboratory, Graduate School of Medical Life Science, Yokohama City University, Yokohama, 230-0045 Japan; 12 Laboratoire de Virologie Moléculaire, Institut National de la Transfusion Sanguine, INSERM U1134, Paris, 75015 France; 13 CREST, JST, Saitama, 332-0012 Japan

## Abstract

Current anti-hepatitis B virus (HBV) agents including interferons and nucleos(t)ide analogs efficiently suppress HBV infection. However, as it is difficult to eliminate HBV from chronically infected liver, alternative anti-HBV agents targeting a new molecule are urgently needed. In this study, we applied a chemical array to high throughput screening of small molecules that interacted with sodium taurocholate cotransporting polypeptide (NTCP), an entry receptor for HBV. From approximately 30,000 compounds, we identified 74 candidates for NTCP interactants, and five out of these were shown to inhibit HBV infection in cell culture. One of such compound, NPD8716, a coumarin derivative, interacted with NTCP and inhibited HBV infection without causing cytotoxicity. Consistent with its NTCP interaction capacity, this compound was shown to block viral attachment to host hepatocytes. NPD8716 also prevented the infection with hepatitis D virus, but not hepatitis C virus, in agreement with NPD8716 specifically inhibiting NTCP-mediated infection. Analysis of derivative compounds showed that the anti-HBV activity of compounds was apparently correlated with the affinity to NTCP and the capacity to impair NTCP-mediated bile acid uptake. These results are the first to show that the chemical array technology represents a powerful platform to identify novel viral entry inhibitors.

## Introduction

Hepatitis B virus (HBV) infection is a major public health problem, with an estimated 240 million carriers worldwide^1^. Chronic HBV infection elevates the risk of hepatocellular carcinoma^2^. The current anti-HBV treatments are mainly based on interferons (IFNs) and nucleos(t)ide analogs. IFNα and its pegylated form (peg-IFNα) modulate host immune response to HBV infection or/and directly inhibit viral replication in hepatocytes. Nucleos(t)ide analogs such as lamivudine, adefovir, entecavir, tenofovir, and telbivudine suppress HBV replication by inhibiting HBV polymerase. These antiviral agents significantly reduce HBV loads in patients, however, there still remain a difficulty in eliminating HBV from infected hepatocytes. Moreover, long-term treatment of some of these nucleos(t)ide analogs often select drug-resistant virus, which leads the reduction in therapeutic efficacy^3^. IFNs also show low tolerability because of serious adverse effects. Thus, further development of anti-HBV agents targeting other viral or host factors essential for viral infection/replication is demanded for improvement of therapeutic outcome.

Lack of a practical and low-cost cell culture systems that support HBV infection has hampered the analysis of the mechanisms underlying HBV infection and hence drug development against HBV^4^. Primary human hepatocytes, primary tupaia hepatocytes, and differentiated HepaRG cells have been mainly used as culture models for HBV infection^4^. Recently, sodium taurocholate cotransporting polypeptide (NTCP), a sodium-dependent bile acid transporter specifically distributed in the liver, has been identified as an HBV entry receptor^5^. This discovery enabled to establish HBV-susceptible cells by overexpressing NTCP in a human hepatocyte-derived cell line, which has greatly accelerated the drug discovery process. To date, bile acids such as tauroursodeoxycholic acid, glycoursodeoxycholic acid and ursodeoxycholic acid, and FDA-approved drugs including cyclosporin A, ezetimibe, and irbesartan, have been reported to inhibit HBV entry^6–12^. We have recently identified a series of HBV entry inhibitors including cyclosporin A, oxysterols, Ro41-5253, vanitaracin A, proanthocyanidin, and SCY995 using cell-based screenings^12–17^. Interestingly, most of the above agents were proved to target NTCP and specifically block HBV infection. These evidences clearly point to NTCP as an attractive target for the development of new anti-HBV agents.

**Figure 1:**
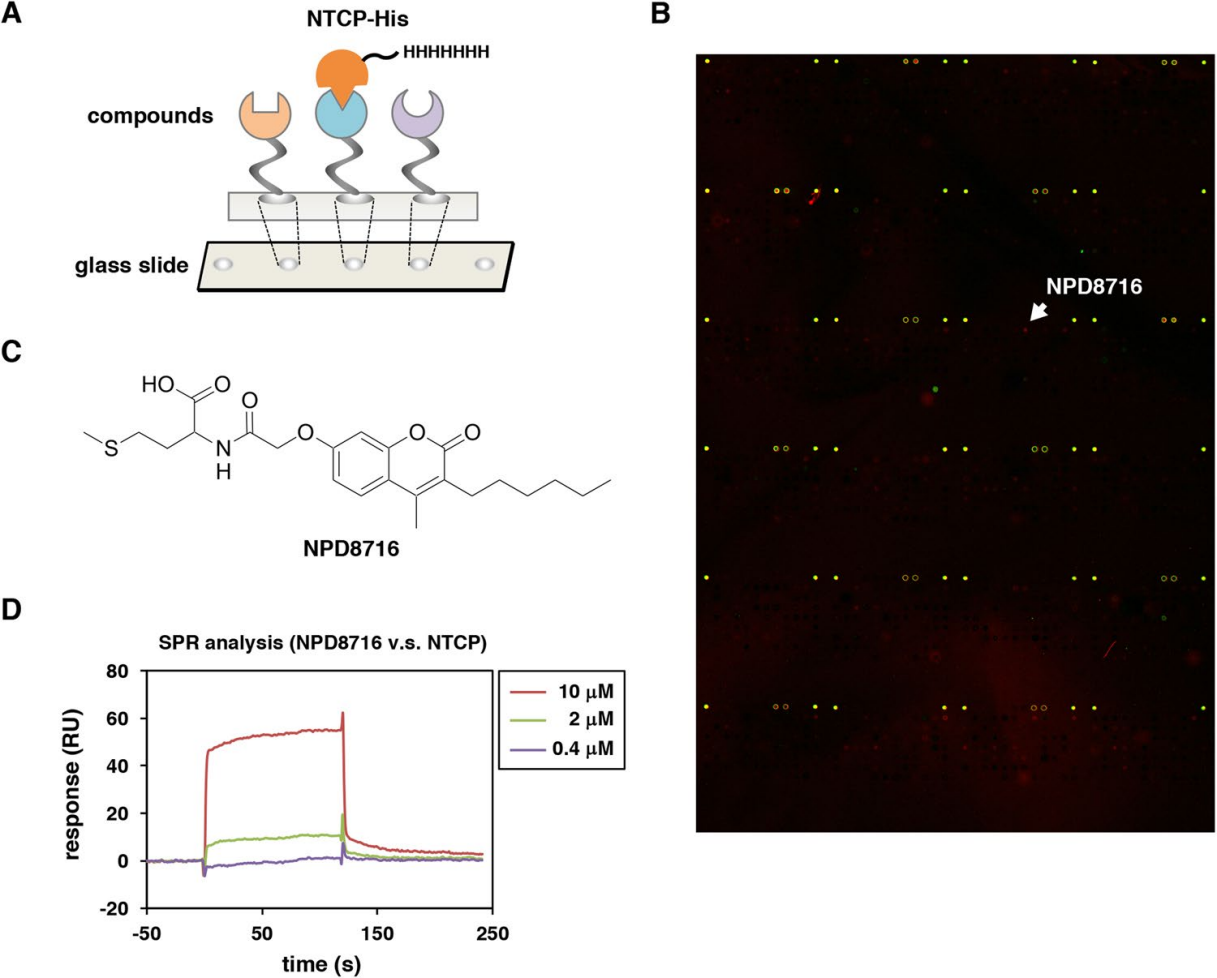
Identification of NTCP-interacting small molecules by chemical array screening. (**A**) Schematic representation of the chemical array screening using recombinant His-tagged NTCP protein (NTCP-His) as a target. The wells immobilized with compounds that can interact with NTCP-His produce fluorescent signals. (**B**) Representative fluorescent image of the chemical array. Positive signal for NTCP interaction is shown by red spot signal (arrow). (**C**) Chemical structure of a coumarin derivative, NPD8716. (**D**) Surface plasmon resonance (SPR) analysis showing the interaction of NPD8716 to NTCP-His. Various concentrations (0.4, 2, or 10 μM) of NPD8716 were injected over a NTCP-His-immobilized sensor chip up to 120 sec as described in Methods. The observed SPR responses are indicated in resonance unit (RU). We identified NPD8716 as one of the candidate compound that interacted with His-NTCP.

Chemical array, in which a panel of small molecules is immobilized on glass slides (Fig. 1A), is a powerful tool for high throughput screening for identifying compounds that interact with a target protein^18^. In this study, we took advantage of the chemical array to identify small molecules interacting with NTCP. From the selected hit compounds, we could identify a novel coumarin derivative inhibiting HBV entry. We further performed a structure-activity relationship analysis using a series of analogs. This study presents a novel methodology to identify viral entry inhibitors using an *in vitro* high throughput screening system.

## Results

### Screening for small molecules that interacted with NTCP

**Figure 2:**
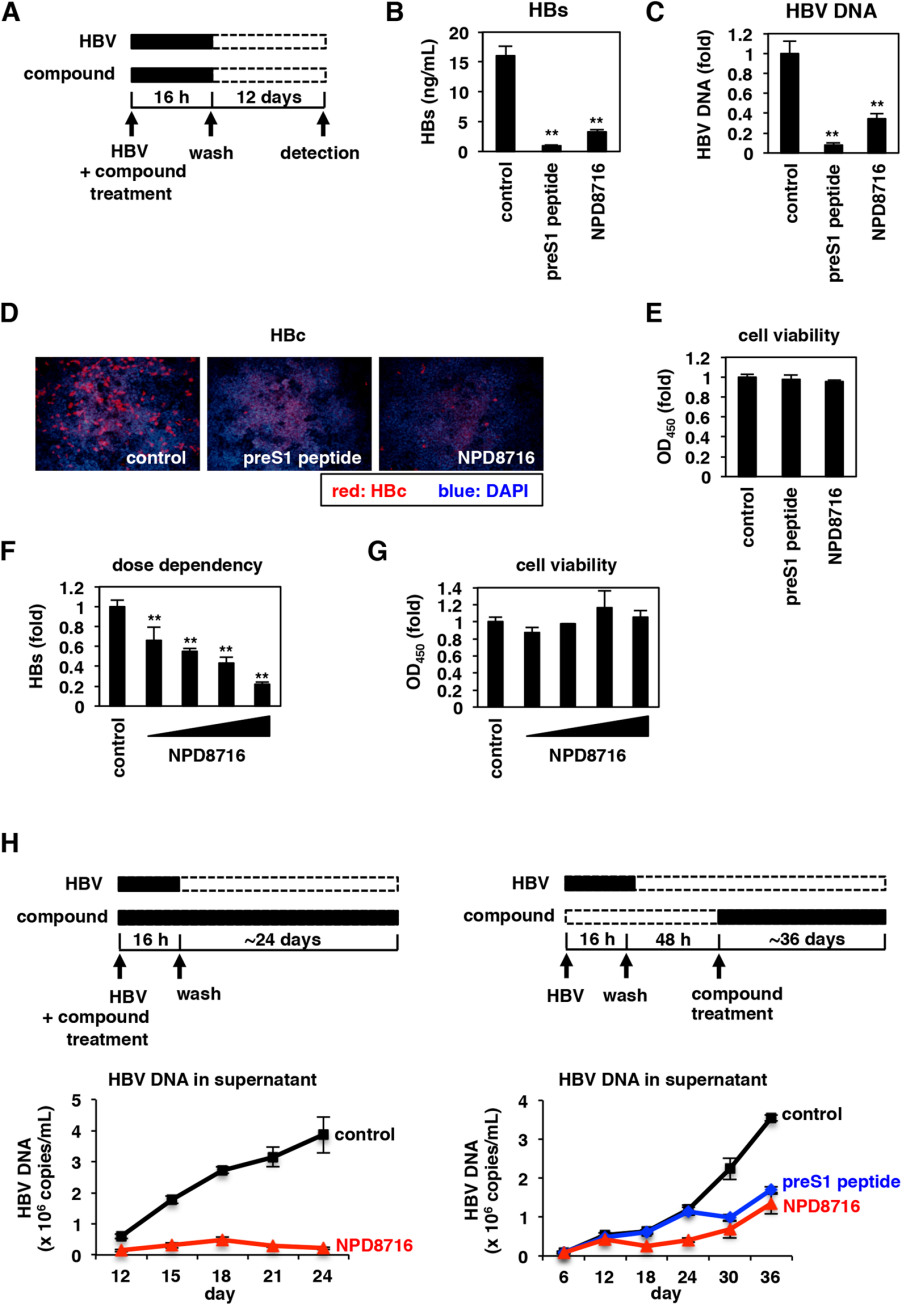
NPD8716 inhibited HBV infection. (**A**) Schematic representation of the scheme for treating the cells with compounds and HBV. HepG2-hNTCP-C4 cells were treated with HBV in the presence or absence of compounds for 16 h. After washing out free HBV and compounds, the cells were cultured without compounds for an additional 12 days, and HBV infection was evaluated by quantifying HBs antigen secreted into the culture supernatant, HBV DNA and HBc antigen in the cells. Black and white bars show periods of treatment and nontreatment, respectively. (**B** to **E**) HepG2-hNTCP-C4 cells inoculated with HBV were treated with or without 100 nM preS1 peptide or 200 μM NPD8716 according to the scheme in (**A**), and HBs antigen in the culture supernatant (**B**), HBV DNA (**C**) and HBc antigen (**D**) in the cells were detected by ELISA, real-time PCR, and immunofluorescence analyses, respectively. Cell viability was also quantified by MTT assay (**E**). Red and blue signals in (**D**) show the detection of HBc antigen and nuclear staining, respectively. (**F**,**G**) HepG2-hNTCP-C4 cells were treated with or without various concentrations (25, 50, 100, or 200 μM) of NPD8716 as shown in (**A**). HBs antigen (**F**) and cell viability (**G**) were determined by ELISA and MTT assay. (**H**) Freshly isolated primary human hepatocytes were continuously treated with or without 60 μM NPD8716 from the time of HBV inoculation (left), or 48 h after HBV inoculation (right). Concentration of NPD8716 was 40 μM at the first three days of treatment and then reduced to 20 μM until day 36, while preS1 peptide was treated at 100 nM in the right panel. At 12, 15, 18, 21, and 24 days (left), or 6, 12, 18, 24, 30, and 36 days (right) postinoculation, HBV DNA secreted into the culture supernatant were detected by real-time PCR (right). SDs are also shown as error bars. Statistical significance was determined by using Student’s *t* test (*P < 0.05; **P < 0.01).

To identify small molecules that inhibit HBV infection, we focused on NTCP as a target molecule and prepared recombinant His-tagged NTCP protein (NTCP-His) as described in Methods. *In vitro* synthesized NTCP-His were, at least partially, functional as NTCP-His bound to its substrate, taurocholic acid, in scintillation proximity assay and interacted with HBV large surface envelope protein (LHBs) in AlphaScreen assay as described previously^12^. The chemical arrays immobilizing 29,707 compounds were probed with the recombinant NTCP-His (Fig. 1A)^18,19^. After washing out free protein, we detected a compound that binds to NTCP-His by immunostaining with anti-His antibody and Cy5-labeled secondary antibody (Fig. 1B). As a result, we observed 74 compounds that showed strong fluorescence spots of NTCP-His (Fig. 1B, red signal). These hit compounds were next subjected to the HBV infection assay in HepG2-hNTCP-C4 cells, an HBV susceptible cell line stably overexpressing NTCP in HepG2 cells, for evaluation of anti-HBV activity according to the method shown in Fig. 2A. Among the 74 primary hits, five compounds were shown to reduce HBV infection to less than 33% of the control (Supplementary Fig. S1). These second hits included two derivatives of bile acids, one of which is a known NTCP substrate, chenodeoxycholic acid^20^, indicating that the chemical array screening successfully captured NTCP-interacting compounds (Supplementary Fig. S1). The low percentage of HBV inhibition by the primary hit compounds (5/74 = 6.8%) is likely to be due to the possibilities including the low stability or rapid metabolism/exclusion of compounds in cell culture system, adsorption of compounds with serum-derived factors such as albumin in the medium that causes inactivation of compounds, and binding to NTCP domains not involved in HBV entry.

In the following analysis, we focused on a coumarin derivative, NPD8716 (Fig. 1C), since coumarin derivatives were reported to inhibit the development of hepatitis and hepatocellular carcinoma^21^. This compound showed a direct interaction with NTCP-His, determined in a surface plasmon resonance (SPR) analysis (Fig. 1D).

### NPD8716 inhibited HBV infection

We examined the effect of NPD8716 on HBV infection. In HBV infection assay, HepG2-hNTCP-C4 cells were inoculated with HBV in the presence or absence of NPD8716 for 16 h (Fig. 2A). A known HBV entry inhibitor, a myristoylated preS1-peptide was used as a positive control^22–24^. After washing out free HBV and compound, and further culture for 12 days, we measured extracellular HBs antigen, intracellular HBV DNA and HBc antigen (Fig. 2B–D) as markers of HBV infection as well as measured cell viability by MTT assay (Fig. 2E). NPD8716 as well as preS1-peptide significantly reduced the HBs level (Fig. 2B). These agents also reduced intracellular HBV DNA (Fig. 2C) and HBc antigen (Fig. 2D), without showing any cytotoxicity (Fig. 2E). Inhibition of HBV infection by NPD8716 was dose-dependent without significant cytotoxicity (Fig. 2F and G). These data suggest that NPD8716 inhibited HBV infection.

Freshly isolated primary human hepatocytes were reported to support HBV spread by production of infectious virions and re-infection into naive cells^25^, which was observed by the amplification of HBV DNA in the culture supernatant of primary human hepatocytes (Fig. 2H, black). However, such HBV amplification was remarkably inhibited upon continuous treatment with NPD8716, either treated from the time of HBV inoculation (Fig. 2H left, red) or after inoculation (Fig. 2H right, red).

### NPD8716 blocked the HBV preS1-mediated attachment to host cells

**Figure 3:**
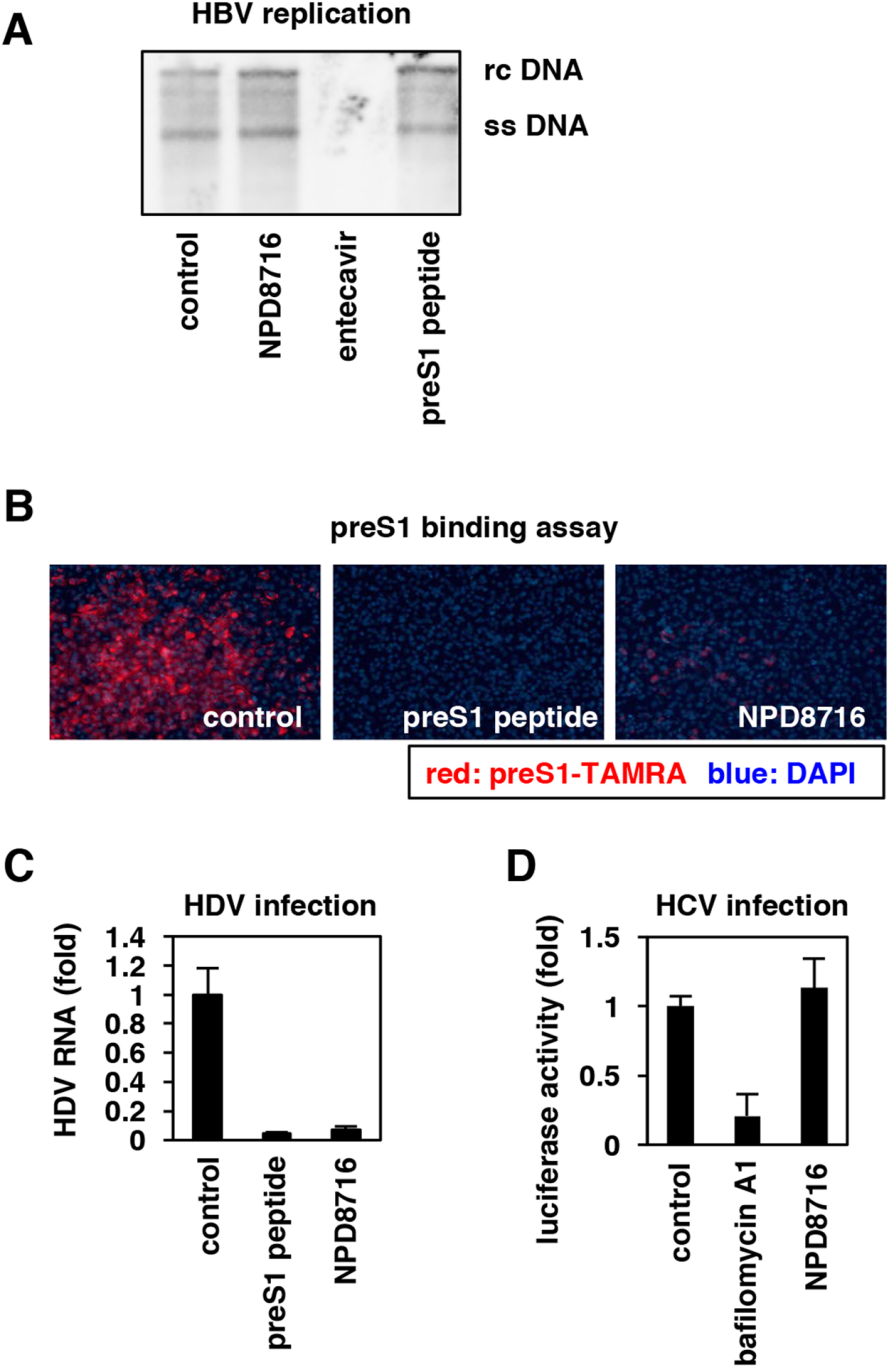
NPD8716 inhibited HBV preS1 attachment to host hepatocytes. (**A**) HBV replication was evaluated by detecting nucleocapsid-associated HBV DNA in Hep38.7-Tet cells treated with or without the indicated compounds (200 μM NPD8716, 100 nM entecavir, 100 nM preS1 peptide) under tetracycline depletion for 6 days by southern blot analysis. rcDNA and ssDNA show HBV relaxed circular DNA and single strand DNA, respectively. (**B**) HBV preS1 attachment to HepG2-hNTCP-C4 cells were evaluated by treating cells with TAMRA-labeled preS1 peptide in the presence or absence of the indicated compounds (100 nM preS1 peptide, or 200 μM NPD8716) for 30 min. Red and blue signals show TAMRA-labeled preS1 peptide and the nucleus, respectively. NPD8716 was suggested to inhibit HBV preS1-mediated host cell attachment. (**C**) Hepatitis D virus (HDV) infection assay. HepG2-hNTCP-C4 cells were inoculated with HDV in the absence or presence of compounds (100 nM pre-S1 peptide or 200 μM NPD8716) as shown in Materials and Methods. HBV infection was evaluated with HDV RNA levels in the cells quantified by real-time RT-PCR analysis. (**D**) Hepatitis C virus (HCV) infection assay. HCV entry was evaluated by HCV pseudoparticle system as described in Methods upon treatment with or without 10 nM bafilomycin A1 or 200 μM NPD8716.

We next investigated which step in the HBV life cycle was blocked by NPD8716. HBV entry into hepatocytes after viral attachment and internalization leads to the formation of covalently closed circular DNA (cccDNA) (Supplementary Fig. S2). This early infection phase is followed by the replication process including transcription, reverse transcription, envelopment, and release (Supplementary Fig. S2). At first, we evaluated the effect of NPD8716 on HBV replication process using Hep38.7-Tet cells, which produce HBV under depletion of tetracycline but do not support the entry phase of infection for lack of NTCP expression^26^. We treated Hep38.7-Tet cells with or without compounds for 6 days in the absence of tetracycline, and then detected nucleocapsid-associated HBV DNA, a replication intermediate, by Southern blot analysis. In contrast to the drastic reduction of nucleocapsid-associated HBV DNA by treatment with entecavir, a clinically available nucleoside analog^2,3^, NPD8716 as well as preS1 peptide had little effect on HBV replication level (Fig. 3A, Supplementary Fig. S3).

We then examined the effect of NPD8716 on viral attachment to host cell surface. It is known that 2–48 aa of the preS1 region of the HBV LHBs envelope protein is essentially involved in the HBV entry process through binding to an entry receptor, NTCP^5,22–24^. A fluorescence-labeled preS1 peptide (2–48 aa) was used to evaluate the preS1-mediated attachment to host cell surface^5,14–16^. As shown in Fig. 3B, NPD8716 drastically reduced the TAMRA-preS1 attachment to cells, as was the case with non-labeled preS1-peptide as a positive control (Fig. 3B). The above results suggest that NPD8716 primarily blocked the entry process by preventing the preS1-NTCP interaction.

This conclusion was further supported by the examination in the infection assay using other hepatitis viruses. Hepatitis D virus (HDV) follows an entry process identical -or very similar- to that is used by HBV in using NTCP as an entry receptor, while hepatitis C virus (HCV) enter into host cells in a different manner, independent of NTCP^27^. As shown in Fig. 3C and D, NPD8716 significantly inhibited the infection of HDV, but not HCV (Fig. 3C and D), suggesting that NPD8716 specifically blocked NTCP-mediated viral entry.

### NPD8716 impaired the NTCP-dependent bile acid uptake

**Figure 4:**
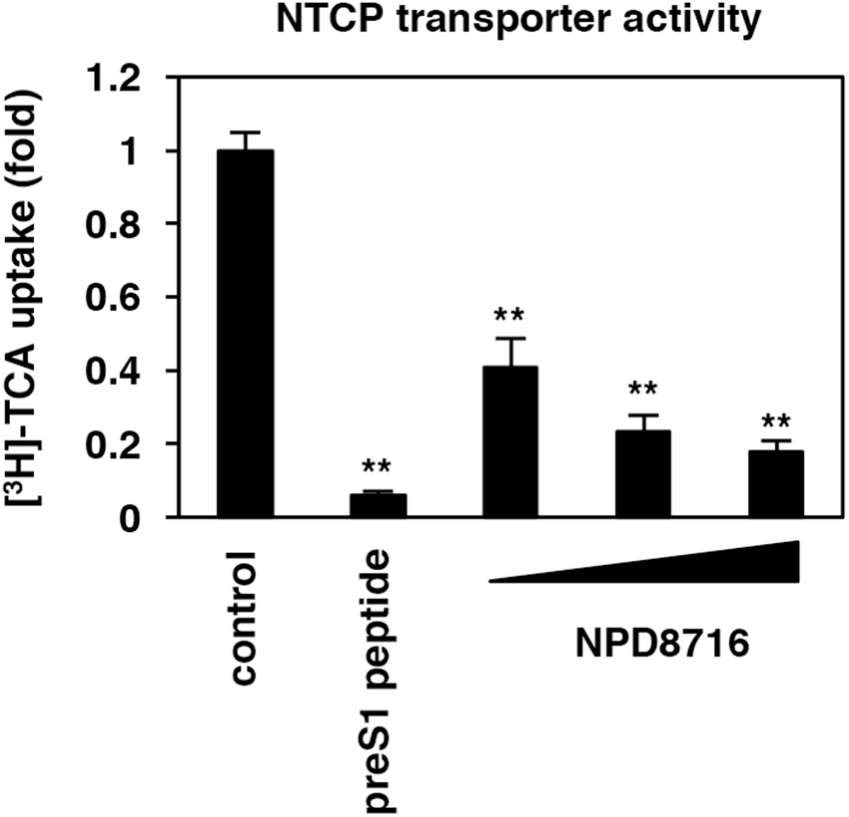
NPD8716 impaired the transporter activity of NTCP. NTCP-dependent bile acid uptake was evaluated by treating HepG2-hNTCP-C4 cells with [^3^H]-taurocholic acid (TCA) in the presence or absence of the indicated compounds (100 nM preS1 peptide, 30, 90, or 270 μM NPD8716) in the sodium-containing buffer as described in Methods.

Most anti-HBV entry inhibitors targeting NTCP reported so far such as preS1 peptide, cyclosporin A, irbesartan, ezetimibe, ritonavir, and vanitaracin A had potentials to impair the NTCP-mediated bile acid uptake^6,7,9–12,14,28^. We then measured the NTCP-mediated uptake of [^3^H]-taurocholic acid (TCA) in HepG2-hNTCP-C4 cells either in the presence or absence of compounds in a sodium-containing condition. As shown in Fig. 4, NPD8716 reduced the NTCP-mediated [^3^H]-TCA uptake in a dose dependent manner (Fig. 4).

### Derivative analysis of NPD8716 for determining structure-activity relationship

**Figure 5:**
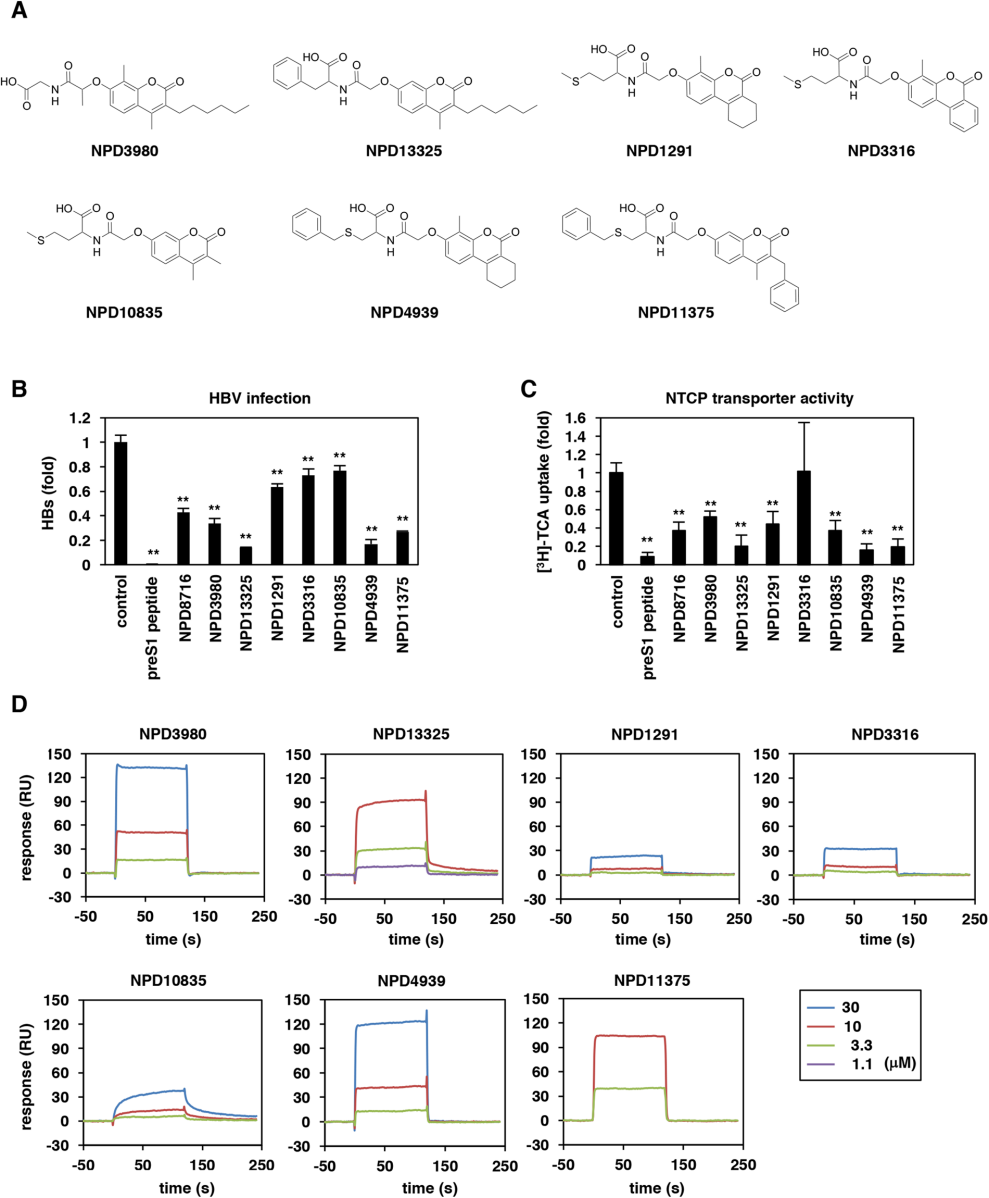
Derivative analysis for the inhibition capacity to HBV infection and to NTCP-mediated bile acid uptake. (**A**) Chemical structures of NPD8716 analogs having significant anti-HBV activities. (**B**) HBV infection to primary human hepatocytes were examined essentially as described in Fig. 2A in the presence or absence of the indicated compounds (100 nM preS1 peptide, 270 μM NPD8716 and its analogs). HBV infection was monitored by detecting HBs antigen in the culture supernatant at 12 days postinfection. (**C**) NTCP-dependent bile acid uptake was evaluated as described in Fig. 4 with or without the indicated compounds (100 nM preS1 peptide, 270 μM NPD8716 and its analogs). (**D**) Kinetics for interaction of NPD8716 analogs to NTCP-His measured by SPR analysis as in Fig. 1D. Various concentrations (1.1, 3.3, 10, or 30 μM for purple, green, red, and blue) of NPD8716 analogs were injected over a sensor chip immobilized with NTCP-His protein.

**Figure 6:**
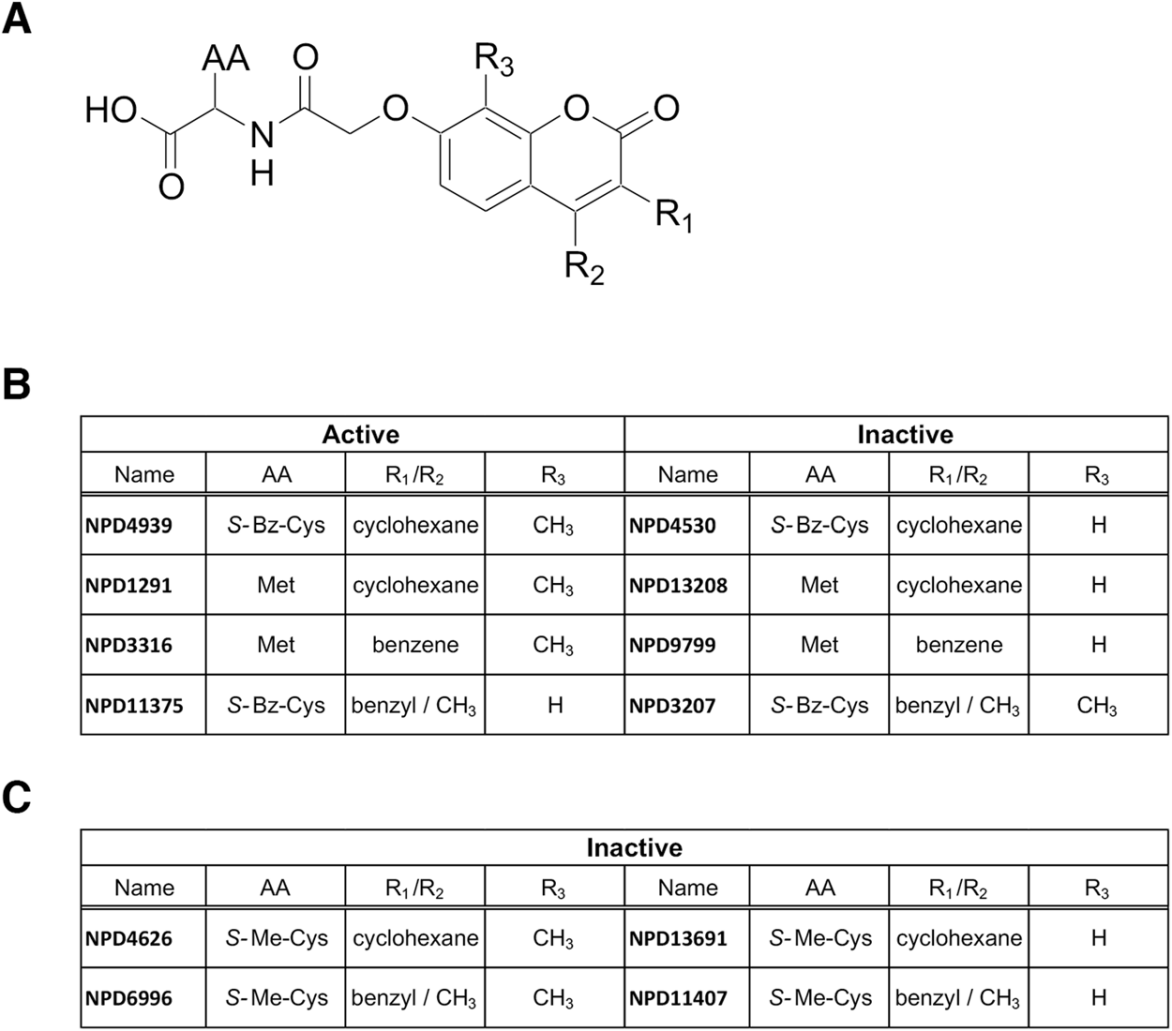
Structure activity relationship analysis. (**A**) The maximum common substructure (MCS) of anti-HBV active compounds. (**B**) Contribution of residual groups for the activity. (**C**) Compounds with S-Met-Cys do not show any activity regardless of R3.

To further investigate the structure essential for the anti-HBV activity, we performed an analog analysis of NPD8716. We tested anti-HBV ability of 46 analogs of NPD8716 in the preS1 binding assay and found that seven analogs out of 46 compounds showed an apparent reduction in the preS1-host cell binding (Table 1). All the seven analogs (Fig. 5A) significantly inhibited HBV infection in primary human hepatocytes (Fig. 5B). We found the maximum common substructure (MCS) of active compounds (Fig. 6A) that include a coumarin scaffold consisting of amino acids via hydroxyl acetyl linker. Furthermore, the residual groups R1, R2, and R3 of coumarin contribute to the anti-HBV activity. For example, in the case of R1/R2 being cyclohexane or benzene ring, the presence or absence of activity mainly depends on R3, i.e. the compounds with methyl group at R3 inhibited the infection (NPD4939, NPD1291, NPD3316 vs NPD4530, NPD13208, NPD 9799), but not vice versa when R1/R2/AA is benzyl/methyl/S-benzyl cysteine (NPD11375 vs NPD3207) (Fig. 6B). Regarding amino acid, the one with an aromatic moiety, such as phenylalanine and S-benzyl cysteine, tended to enhance the anti-HBV activity (NPD11375, NPD4939, NPD13325). Furthermore, S-methyl cysteine must be unfavorable, because none of the compounds having that AA (NPD4626, NPD6996, NPD13691, and NPD11407) demonstrated an activity regardless of R3 group (Fig. 6C). Interestingly, the capacity to inhibit HBV entry was apparently correlated with the activity to impair NTCP-mediated bile acid uptake (Fig. 5C), and with the binding response to NTCP protein (Fig. 5D). These results are consistent with the previous report suggesting that the NTCP’s viral receptor function shares the common determinants on the bile acid transporter activity^20^. The 50% inhibitory concentration (IC_50_) for HBV infection and bile acid transport, and the 50% cytotoxic concentration (CC_50_) are shown in Table 2. This analog campaign identified compounds with higher anti-HBV potency such as NPD4939.

**Table 1:** Structure and anti-HBV activity of NPD8716 analogs. Anti-HBV activity was evaluated by preS1 binding assay and is shown by “+” or “—”.

name	structure	inhibition of preS1 attachment	name	structure	inhibition of preS1 attachment
NPD733		—	NPD4003		—
NPD1316		—	NPD4067		—
NPD1285		—	NPD4385		—
NPD1331		—	NPD3980		+
NPD1401		—	NPD8637		—
NPD1743		—	NPD8716		+
NPD1764		—	NPD10403		—
NPD641		—	NPD10857		—
NPD780		—	NPD10934		—
NPD615		—	NPD10957		—
NPD1396		—	NPD10969		—
NPD1158		−	NPD11356		—
NPD13324		—	NPD10962		—
NPD13325		+	NPD12312		—
NPD1291		+	NPD13208		—
NPD1582		—	NPD3207		—
NPD1835		—	NPD4530		—
NPD1737		—	NPD4626		—
NPD4447		—	NPD4939		+
NPD3316		+	NPD6996		—
NPD8798		—	NPD11375		+
NPD9799		—	NPD11407		—
NPD10673		—	NPD13691		—
NPD10835		+

**Table 2:** Chemical structures and bioactivities of NPD8716 and its analogs. IC_50_s for inhibiting HBV infection and NTCP-mediated bile acid uptake are shown on the second and the third columns, respectively. CC_50_s determined by MTT assay are also shown in the fourth column. IC_50_s and CC_50_s are shown in μM.

name & structure	IC_50_ (HBV infection)	IC_50_ (NTCP transporter)	CC_50_
NPD8716	238 ± 23.6	17.8 ± 1.1	>270
NPD3980	194 ± 22.7	34.9 ± 12.3	>270
NPD13325	145 ± 9.5	12.5 ± 1.5	>270
NPD4939	123 ± 7.4	16.2 ± 1.4	>270
NPD11375	150 ± 11.3	13.3 ± 1.9	>270

## Discussion

In this study, through *in vitro* screening using chemical array, we identified novel small molecules that bound to NTCP and inhibited HBV entry. So far, the chemical array system has been applied to identify compounds that interact with physiologically essential molecules including carbonic anhydrase II, MDM2, MTH1, trichothecene 3-O-acetyltransferase, and matrix metalloproteinase that may serve as leads for inhibitors of glaucoma, carcinogenesis, mycotoxin, and cancer metastasis^29–33^. This system also identified compounds interacting with influenza virus nucleoprotein and HIV-1 Vpr^34,35^. The advantage of this method is to be able to identify candidate compounds in a single *in vitro* assay with a small amount of target protein^36^. In general, virus entry into host cell is an attractive drug target because viral entry is essential for initiating, spreading, and maintaining virus infection^27^. In this study, we used an HBV entry receptor, NTCP, as a target molecule and identified coumarin derivatives as HBV entry inhibitors. To date, coumarin derivatives are clinically used in safe including an anticoagulant, warfarin, and antibiotics, novobiocin and coumermycin, which raises this class of compounds as potential candidates in drug development. Further derivative analysis is expected to identify more potent anti-HBV agents, which is a future subject. It would be also interesting to know whether coumarins inhibit only HBV cell free infection or also possible cell-to-cell transmission of HBV, if any.

HBV entry inhibitors are useful to block de novo infection that causes a rapid spread of HBV infection: They are primarily expected to be useful for prevention of HBV recurrence after liver transplantation and of vertical transmission, and as a post-exposure prophylaxis. In addition, HBV entry inhibitors may be beneficial to treat chronic hepatitis B, as myrcludex-B, an HBV entry inhibitor, had a potential to reduce HBV in already infected patients^37,38^. Moreover, in contrast to myrcludex-B, which is not orally bioavailable and immunogenic because of its nature of a peptidic agent, small molecules identified in this study such as NPD8716 can potentially overcome these problems. This is the first report demonstrating the usefulness of the chemical array technology for identifying inhibitors of viral entry process. Thus, chemical array will offer a powerful platform to identify new drug candidates against infectious diseases. Further in-depth studies including derivative analysis and *in vivo* infection experiments are expected to present a new class of anti-HBV agents.

## Methods

### Reagents

All the compounds used for screening were supplied from the RIKEN Natural Products Depository, NPDepo^18,19^. The purity and structures of NPD8716 and its derivatives were confirmed by LC-MS analysis. A preS1 peptide, consisting of 2–48 aa of the HBV preS1 region, and TAMRA-labeled preS1 peptide were synthesized (CS bio and Scrum, Inc.). Entecavir was purchased from Santa Cruz Biotechnology. Bovine serum albumin (BSA) was obtained from Wako.

### Purification of recombinant NTCP protein

His-tagged human recombinant NTCP protein (NTCP-His) was purified using a wheat germ cell free system or *E. coli* expression system essentially described previously^12,14,15^. The purity of the protein was 50–80%, determined by Coomassie Brilliant Blue staining^15^. The recombinant NTCP-His, at least in part, was suggested to be active from the results in scintillation proximity assay and AlphaScreen assay as already described^12^.

### Chemical array screening

Small molecules interacting with recombinant NTCP protein was screened by chemical arrays as described previously^30,32^. Briefly, the slides with 29,707 immobilized compounds were probed at 30 °C for 1 h with 1 μM NTCP-His. After washing, the probed slides were incubated with anti-His antibody (mouse IgG, 1/1000 diluted, GE Healthcare), followed by another incubation with a Cy5-conjugated second antibody (goat anti-mouse IgG, 1/1000 diluted, Millipore) at 30 °C for 1 h. The slides were scanned with a GenePix microarray scanner (Molecular Devices) using the Cy5 channel.

### Cell culture

HepG2-hNTCP-C4, Hep38.7-Tet cells, and primary human hepatocytes (Phoenixbio) were cultured as described previously^13,14,26^.

### HBV preparation and infection

HBV inoculum used in this study was mainly prepared from the supernatant of Hep38.7-Tet cells (genotype D) cultured in the absence of tetracycline as described^26^. In the HBV infection assay, HBV was inoculated at 12,000 genome equivalents (GEq)/cell in the presence of 4% polyethylene glycol 8000 (PEG 8000) to HepG2-hNTCP-C4 or primary human hepatocytes for 16 h, as described previously^39^.

### Detection of HBs antigens

HBs antigen produced in the culture supernatant was quantified by ELISA essentially as described previously^39^.

### Detection of HBV DNA

HBV DNA was quantified by real time PCR analysis using the primer set 5′-ACTCACCAACCTCCTGTCCT-3′ and 5′-GACAAACGGGCAACATACCT-3′ and probe 5′-carboxyfluorescein (FAM)-TATCGCTGGATGTGTCTGCGGCGT- carboxytetramethylrhodamine (TAMRA)-3′^12^.

### Southern blot analysis

Nucleocapsid-associated DNA was extracted by digestion of free nucleic acids in cell lysate with DNase I and RNase A, followed by treatment with proteinase K as previously described^39^. Southern blot analysis to detect HBV DNAs were performed as previously described^39^.

### Indirect immunofluorescence analysis

Indirect immunofluorescence analysis was performed essentially as described previously using an anti-HBc antibody (Thermofisher)^39^.

### MTT assay

The MTT cell viability assay was performed as described previously using Cell Proliferation Kit II XTT (Roche)^26^.

### HBV replication assay

HBV replication was examined using Hep38.7-Tet cells in which HBV replication was induced by depletion of tetracycline for 6 days as described previously^39^. Nucleocapsid-associated HBV DNA in the cells were detected by Southern blot.

### PreS1 peptide binding assay

PreS1-mediated attachment to host hepatocytes was examined by treating HepG2-hNTCP-C4 cells with 40 nM TAMRA-labeled preS1 peptide, spanning 2–48 amino acids of the preS1 region (preS1-TAMRA) for 30 min at 37 °C, essentially as previously described^5,14–16^.

### Surface plasmon resonance analysis

The binding kinetics of compounds to NTCP proteins was measured by a Biacore X100 instrument (GE Healthcare). Recombinant NTCP protein^14,15^ was immobilized onto the surface of a CM5 sensor chip using an amine coupling kit (GE Healthcare). Different concentrations of compounds diluted with HBS-EP (10 mM HEPES, 150 mM NaCl, 3 mM EDTA, 0.005% surfactant P20, pH7.4) containing 5% DMSO were injected onto the sensor chip at a flow rate of 20 μl/min for 120 sec at 25 °C, followed by applying a compound-free buffer for 120 sec. The bulk effect of DMSO was subtracted using reference flow cells. Kinetic parameters were determined by analyzing the data using Biacore X100 Evaluation software (GE Healthcare).

### NTCP transporter assay

HepG2-hNTCP-C4 cells were incubated with [^3^H]-taurocholic acid in the presence or absence of compounds in a sodium-containing buffer at 37 °C for 15 min to allow substrate uptake into cells. The cells were then washed and lysed to measure the intracellular radioactivity by liquid scintillation counter.

### HCV infection assay

HCV envelope-dependent infection was examined by HCV pseudoparticle system (kindly provided by Dr. Francois-Loic Cosset at the Universite de Lyon) in Huh-7.5.1 cells as described previously^14^.

### HDV infection assay

HDV was recovered from the culture supernatant of Huh-7 cells transfected with pT7HB2.7 and pSVLD3 (kindly provided by John Taylor at the Fox Chase Cancer Center)^14^. HDV infection was examined by treating HepG2-hNTCP-C4 cells with HDV at 15 GEq/cell in 5% PEG8000 as described previously^14^.

### Statistics

Statistical significance was determined using Student’s t-test (*P < 0.05, **P < 0.01).

## Electronic supplementary material

Supplementary Information
