# Supplementary material for: Chemical array system, a platform to identify novel hepatitis B virus entry inhibitors targeting sodium taurocholate cotransporting polypeptide
Source: Sci Rep. 2018 Feb 9;8:2769. doi: 10.1038/s41598-018-20987-w (PMC5807303; doi:10.1038/s41598-018-20987-w)
Supplement: Supplementary file 1 — Supplementary Information [file 41598_2018_20987_MOESM1_ESM.pdf]

[Supplementary Information]

Chemical array system, a platform to identify novel hepatitis B virus entry inhibitors targeting sodium taurocholate cotransporting polypeptide

Manabu Kaneko[1,2+], Yushi Futamura[3+], Senko Tsukuda[1,4], Yasumitsu Kondoh[3], Tomomi Sekine[5], Hiroyuki Hirano[6], Kento Fukano[1,7], Hirofumi Ohashi[1,2], Wakana Saso[1,8], Ryo Morishita[9], Satoko Matsunaga[10], Fumihiro Kawai[11], Akihide Ryo[10], Sam-Yong Park[11], Ryosuke Suzuki[1], Hideki Aizaki[1], Naoko Ohtani[2], Camille Sureau[12], Takaji Wakita[1], Hiroyuki Osada[3¶]*, Koichi Watashi[1,2,13¶]*

[1]Department of Virology II, National Institute of Infectious Diseases, Tokyo, 162-8640, Japan, [2]Department of Applied Biological Sciences, Tokyo University of Science, Noda, 278-8510, Japan, [3]Chemical Biology Research Group, RIKEN Center for Sustainable Resource Science (CSRS), Wako, 351-0198, Japan, [4]Micro-signaling Regulation Technology Unit, RIKEN Center for Life Science Technologies (CLST), Wako, 351-0198, Japan, [5]Bio-Active Compounds Discovery Research Unit, RIKEN CSRS, Wako, 351-0198, Japan, [6]Chemical Resource Development Research Unit, RIKEN CSRS, Wako, 351-0198, Japan, [7]Department of Analytical Biochemistry, Meiji Pharmaceutical University, Kiyose, 204-8588, Japan, [8]The Institute of Medical Science, The University of Tokyo, Tokyo, 108-8639, Japan, [9]CellFree Sciences Co., Ltd., Matsuyama, 790-8577, Japan, [10]Department of Microbiology, Yokohama City University Graduate School of Medicine, Yokohama, 236-0027, Japan, [11]Drug Design Laboratory, Graduate School of Medical Life Science, Yokohama City University, Yokohama, 230-0045, Japan, [12]Laboratoire de Virologie Moléculaire, Institut National de la Transfusion Sanguine, INSERM U1134, Paris, 75015, France, [13]CREST, JST, Saitama, 332-0012, Japan.

[+,¶] These authors contributed equally to this work.

*Address correspondence to:
Koichi Watashi, Ph.D.
Department of Virology II, National Institute of Infectious Diseases
1-23-1 Toyama, Shinjuku-ku, Tokyo, 162-8640, Japan
E-mail: kwatashi@nih.go.jp; Tel: +81-3-5285-1111; Fax: +81-3-5285-1161

Hiroyuki Osada, Ph.D.
Chemical Biology Research Group, RIKEN CSRS
2-1 Hirosawa, Wako, Saitama 351-0198, Japan
E-mail: hisyo@riken.jp; Tel: +81-48-467-9541; Fax: +81-48-462-4669

Supplementary Figure

Fig. S1

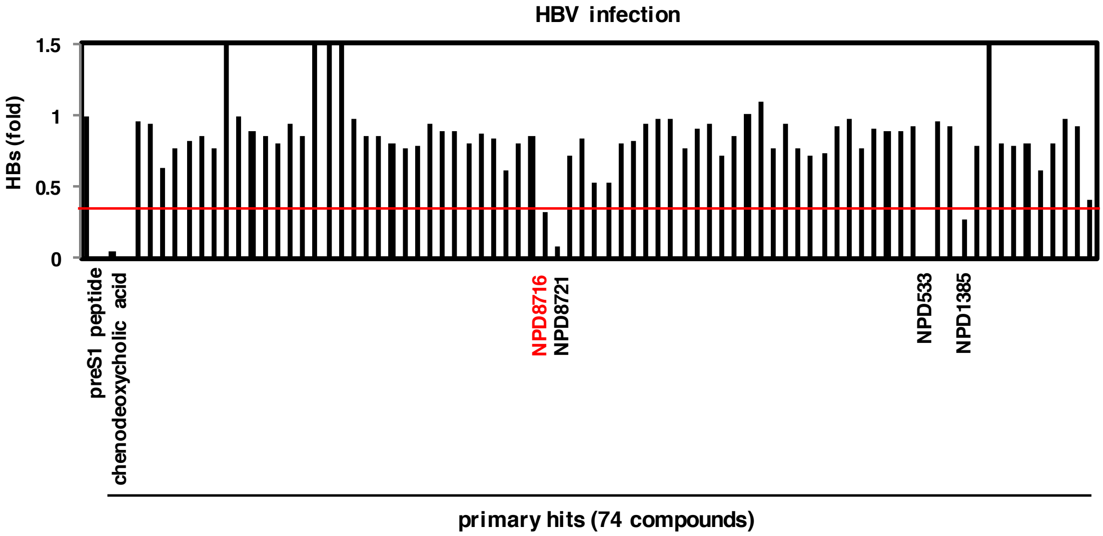

Fig. S1. Screening of the primary hit compounds in HBV infection assay. HepG2-hNTCP-C4 cells were treated with HBV in the presence or absence of compounds (100 μg/mL) according to the scheme in Fig. 2A, and HBs antigen in the culture supernatant was detected by ELISA. Five compounds reduced the infection to less than 33% (red bar). Chenodeoxycholic acid and NPD1385 are bile acid analogs. This study focuses on one of the hits, NPD8716, shown in red.

Fig. S2

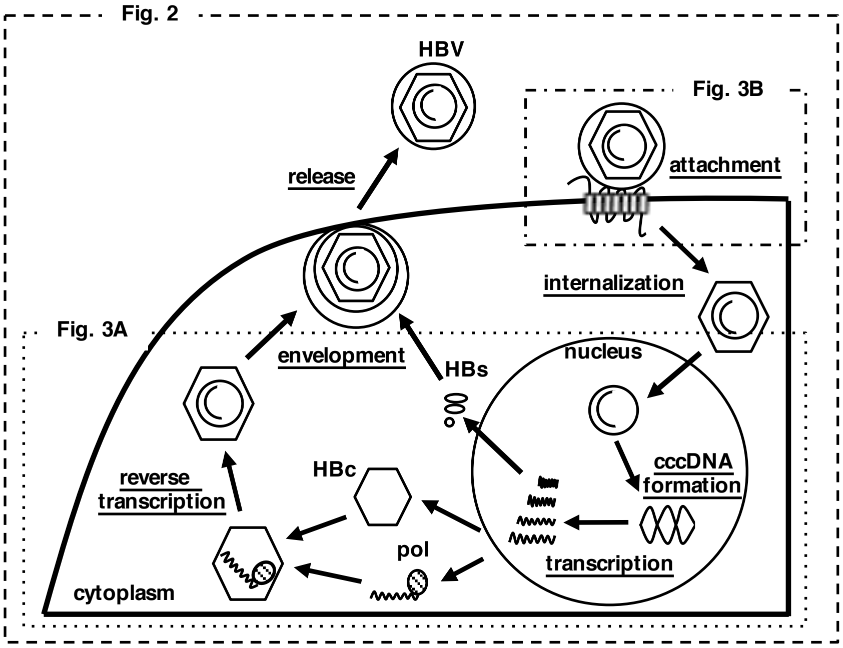

Fig. S2. Schematic representation of the HBV life cycle. A summary of the HBV life cycle is described in the Results and Discussion section. The assays shown in Fig. 2, Fig. 3A, and Fig. 3B evaluate the whole life cycle, the replication process, and the viral attachment, respectively.

Fig. S3

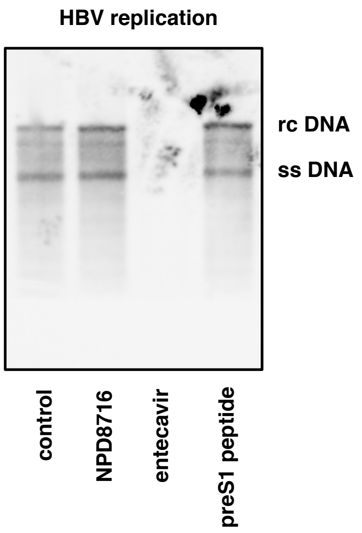

Fig. S3. The raw data of the Southern blot shown in Fig. 3A.
